# The Relationship between Glymphatic Dysfunction and Post-stroke Cognitive Impairment

**DOI:** 10.2174/0115734056433163251027052013

**Published:** 2025-11-28

**Authors:** Hongjie Huang, Hongqian Tian, Zihuai Fang, Zitong Min, Mingyang Peng, Mingxu Jin, Liang Jiang, Xindao Yin

**Affiliations:** 1 Department of Radiology, Nanjing First Hospital, Nanjing Medical University, Nanjing 210006, China; 2 Department of Radiology, Nanjing Yuhua Hospital, Nanjing 210000, China

**Keywords:** Post-stroke cognitive impairment, Glymphatic system, Diffusion tensor imaging, Perivascular space, Choroid plexus volume, ALPS index

## Abstract

**Background:**

Glymphatic dysfunction is proposed as a final common pathway to dementia. Cognitive impairment following ischemic stroke can gradually worsen, potentially leading to post-stroke dementia. This study aimed to examine the changes in glymphatic function in post-stroke patients and explore its relationship with cognition.

**Methods:**

A total of thirty-two post-stroke patients and twenty-seven healthy controls (HCs) matched for age, sex, and educational level were enrolled in this study. All participants underwent neurological MRI scans and comprehensive cognitive assessments six months following the onset of the stroke. Three glymphatic markers derived from MRI were quantified, including diffusion tensor image analysis along the perivascular space (DTI-ALPS) index, choroid plexus volume (CPV), and enlarged perivascular spaces (PVS) volume. The changes in glymphatic markers and their correlations with cognitive scores were analyzed.

**Results:**

Post-stroke patients exhibited a significantly decreased DTI-ALPS index (*p* < 0.001) and an increased CPV (*p* < 0.001) compared to HCs, while no significant difference was observed in PVS volume. Correlation analysis revealed that the DTI-ALPS index was positively correlated with Digit Span Test (*r* = 0.426, *p* = 0.015) and Digit Symbol Substitution Test (*r_s_* = 0.363, *p* = 0.041) scores, and PVS volume showed a positive correlation with Trail Making Test-B scores (*r_s_* = 0.391, *p* = 0.027). After adjusting for confounding factors, multiple linear regression analyses indicated that enlarged PVS volume was independently associated with worse performance in Trail Making Test-B (*β* = 0.428, *p* = 0.010).

**Discussion:**

The findings demonstrated that glymphatic dysfunction, as indicated by a reduced DTI-ALPS index and increased CPV volume, was evident in post-stroke patients and significantly linked to impairments in specific cognitive domains, including working memory, processing speed, and executive function. These observations supported the hypothesis that glymphatic impairment may represent a key mechanistic pathway underlying post-stroke cognitive impairment (PSCI). To further elucidate the causal relationships and identify potential therapeutic targets, future studies incorporating larger cohorts, longitudinal designs, and region-specific PVS analyses are warranted.

**Conclusion:**

Post-stroke patients exhibited a reduced DTI-ALPS index and an increased CPV, potentially reflecting impaired glymphatic function. Furthermore, these metrics were associated with specific cognitive domains.

## 1 INTRODUCTION

Stroke is one of the leading causes of vascular cognitive impairment and dementia [1]. Post-stroke cognitive impairment (PSCI) occurs in up to 50% of patients with acute ischemic stroke and may progress to post-stroke dementia, imposing a substantial social and economic burden [1, 2]. However, the underlying mechanisms of PSCI remain largely unclear.

The glymphatic system has recently been recognized as a vital dynamic clearance pathway in the central nervous system [3]. Within the glymphatic pathway, cerebrospinal fluid (CSF) enters the brain *via* periarterial spaces, mixes with interstitial fluid (ISF) in the parenchyma through perivascular astrocytic aquaporin-4 (AQP-4) channels, and then drives the clearance of ISF and solutes along perivenous pathways [4]. Multiple pathological conditions can disrupt glymphatic clearance, initiating a self-reinforcing cascade of protein aggregation, neuroinflammation, and neuronal loss that amplifies glymphatic impairment, culminating in dementia progression through a pathological feed-forward loop [5]. In recent years, various MRI techniques have been widely applied in research to indirectly reflect the glymphatic-related changes in humans. First, diffusion tensor image analysis along the perivascular space (DTI-ALPS), which quantifies water diffusivity along the perivascular spaces (PVS) surrounding the deep medullary veins at the level of the lateral ventricle body [6], showed a significant correlation with glymphatic clearance as assessed by the gold-standard intrathecal gadolinium technique [7]. Second, the choroid plexus contributes to the glymphatic system by producing CSF. Enlargement of the choroid plexus volume (CPV) has been associated with cognitive decline in patients with Alzheimer's disease (AD) [8, 9] and Parkinson's disease (PD) [10]. Third, as a critical component of the glymphatic system, PVS serves as a common approach for evaluating glymphatic function, with significant PVS enlargement being indicative of impaired drainage with the glymphatic system [11-13].

Several animal experiments have confirmed that ischemic stroke can result in glymphatic system dysfunction, which is closely associated with the formation of brain edema [14, 15]. A recent clinical study further used the decreased DTI-ALPS index to reveal glymphatic dysfunction in patients with ischemic stroke [16]. However, research on the relationship between glymphatic dysfunction and cognitive impairment in post-stroke patients remains limited.

Therefore, in this study, we aimed to investigate the changes in glymphatic markers in post-stroke patients to reflect glymphatic function and further analyze the association between glymphatic dysfunction and cognitive decline. We hypothesized that post-stroke patients would exhibit a correlation between glymphatic dysfunction and cognitive decline.

## 2 METHODS

### 2.1 Participants

In this study, 32 post-stroke patients and 27 healthy controls (HCs) were recruited from Nanjing First Hospital between August 2020 and August 2023. The inclusion criteria were as follows: (1) age ≥ 40 years and years of education ≥ 6 years; (2) acute ischemic stroke confirmed by MRI or CT, with a time interval of 5.5-6.5 months from symptom onset to enrollment; (3) no severe aphasia, visual or hearing impairment, and the ability to cooperate with cognitive assessments. The exclusion criteria were as follows: (1) presence of dementia, neurodegenerative disease, or severe mental illness before stroke onset; (2) hemorrhagic stroke or other stroke types; (3) contraindications for MRI or other brain lesions (*e.g*., tumor, encephalitis); (4) long-term use of medications affecting cognition. The sample size for this study was determined based on previous neuroimaging studies that have demonstrated the ability to detect significant glymphatic alterations in comparable patient populations, and was limited by the availability of eligible participants who met our stringent inclusion criteria during the study period.

### 2.2 Neuropsychological Assessments

Cognitive function was systematically evaluated using a standardized neuropsychological test battery encompassing five core domains: global cognition, memory, attention, processing speed, and executive function. The assessment protocol included the following validated instruments: global cognitive status was assessed using the Montreal Cognitive Assessment (MoCA) [17] and the Mini-Mental State Examination (MMSE) [18]. Memory function was evaluated *via* the Auditory Verbal Learning Test (AVLT), which incorporated immediate recall and a 20-minute delayed recall trial (AVLT-D) to assess short-term retention and long-term memory consolidation [19]. Working memory capacities and attention were examined using the Digit Span Test (DST) [20], which featured forward and backward recall conditions. Processing speed was assessed using the Digit Symbol Substitution Test (DSST) [20], which measured visuomotor coordination and psychomotor speed. The Trail Making Test (TMT) [21] was administered in two parts: part A (TMT-A) for assessing visual scanning and processing speed, and part B (TMT-B) for evaluating higher-order executive functions, including cognitive flexibility and task-switching ability.

### 2.3 MRI Acquisition

MRI scans were performed on a 3.0 T MRI scanner (MAGNETOM Prisma, Siemens Healthcare) with a 64-channel head RF coil. The scanning protocol consisted of three-dimensional T1-weighted imaging (3D-T1WI) and diffusion tensor imaging (DTI). The detailed parameters were as follows: 3D-T1WI sequence: sagittal acquisition, repetition time (TR) = 1600 ms, echo time (TE) = 2.3 ms, inversion time (TI) = 900 ms, field of view (FOV) = 240 mm × 240 mm, slice thickness = 0.94 mm, acquisition matrix = 256 × 256, flip angle = 8°; DTI sequence: axial acquisition, TR = 3600 ms, TE = 68 ms, FOV = 220 mm × 220 mm, slice thickness = 4.0 mm, acquisition matrix =128 ×128, flip angle=90°, 30 diffusion-sensitizing gradient directions, b value=1000 s/mm^2^.

### 2.4 Quantification of DTI-ALPS Index

**Fig. (1):**
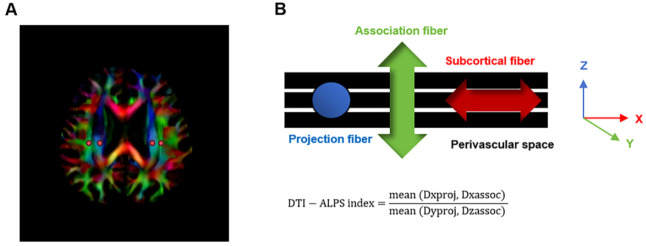
(**A**) The color-coded FA map shows spherical ROIs placed, respectively, on the projection fibers and the association fibers. (**B**) Schematic diagram of DTI-ALPS index calculation.

The DTI-ALPS index was calculated according to the previous study [7]. Initially, DTI data were preprocessed using MRtrix3 (https://www.mrtrix.org) and FSL (https://fsl.fmr ib.ox.ac.uk/fsl/fslwiki). The preprocessing steps included Gibbs ringing artifact removal, eddy current correction, head motion correction, bias field correction, and skull stripping. Subsequently, fractional anisotropy (FA) maps and diffusivity maps along the X-axis (Dx), Y-axis (Dy), and Z-axis (Dz) were generated using FSL. These diffusivity maps were then co-registered to the FA template (JHU-ICBM-FA). We employed an atlas-based approach to define the regions of interest (ROIs). Utilizing the JHU-ICBM-DTI-81-white-matter labeled atlas, we identified the projection fibers and association fibers as the superior corona radiata (SCR) and superior longitudinal fasciculus (SLF) at the level of the lateral ventricular body. Spherical ROIs with a diameter of 5 mm were automatically delineated in the bilateral SCR and SLF regions on the diffusion maps of all subjects. The center coordinates of the ROIs are as follows: left SCR (116,110,99), left SLF (128,110,99), right SCR (64,110,99), and right SLF (51,110,99), based on the JHU-ICBM-FA template. Subsequently, we performed visual inspection of each participant's automatically generated ROI locations using MRIcroGL software (https://www.nitrc.org/projects/mricrogl). To ensure precise ROI placement on the targeted white matter tracts rather than lesioned tissues, we specifically excluded data from participants whose ROIs overlapped with infarction areas, thereby guaranteeing the accuracy of subsequent diffusion metric analyses. Finally, the diffusivities in the directions of the X-axis, Y-axis, and Z-axis were obtained. The DTI-ALPS index was calculated using the following formula: DTI-ALPS index = mean (Dxproj, Dxassoc)/mean (Dyproj, Dzassoc). Here, Dxproj denotes the diffusivity of projection fibers along the X-axis, Dxassoc denotes the diffusivity of association fibers along the X-axis, Dyproj denotes the diffusivity of projection fibers along the Y-axis, and Dzassoc denotes the diffusivity of association fibers along the Z-axis (Fig. **1**). The mean DTI-ALPS index was subsequently calculated as the average of the left and right DTI-ALPS index.

### 2.5 CPV Segmentation

To achieve precise segmentation of the choroid plexus, we employed a fully convolutional neural network model (https://github.com/hettk/chp_seg) for automated segmentation of T1-weighted images [22]. Thereafter, the volumes of the left and right choroid plexus were calculated for each participant. The schematic diagram of CPV segmentation is shown in Fig. (**2**). In the subsequent statistical analysis, all volume measurements were normalized by total intracranial volume to minimize the influence of inter-individual variations in brain size on the study outcomes.

### 2.6 PVS Segmentation

We implemented a deep learning algorithm proposed by Boutinaud *et al*., which is based on an autoencoder and a U-shaped network (U-net) [23]. By using T1-weighted MRI data, we successfully achieved precise segmentation of three-dimensional PVS in the deep white matter (DWM) and basal ganglia (BG). The segmentation result was a probability map with voxel values ranging from 0 to 1. In this study, a threshold of 0.5 was selected to generate the final segmentation result. This threshold was determined through developer validation and visual assessment, aiming to balance detection accuracy and sensitivity. The schematic diagram of PVS volume segmentation is shown in Fig. (**3**). The PVS volume was normalized by the total intracranial volume in the same manner as CPV.

### 2.7 Statistical Analysis

SPSS 27.0 statistical software was used for statistical analysis. Continuous variables have been presented as mean ± standard deviation (SD) for normally distributed data and as median [interquartile range (IQR)] for non-normally distributed data. Categorical variables have been expressed as counts and percentages (n, %). Differences between the two groups were compared using independent-samples t-tests for continuous variables with normal distribution or Mann-Whitney U-tests for continuous variables with non-normal distribution, and χ^2^ tests were used for categorical variables. Correlations between glymphatic markers and cognitive scores were analyzed using Pearson's correlation coefficient for normally distributed variables or Spearman's rank correlation coefficient for non-normally distributed variables. Variables with *p* < 0.05 were further included in the multiple linear regression model. A two-tailed *p*-value of less than 0.05 was considered statistically significant. Given the exploratory nature of this study, the *p*-value was reported without correction for multiple comparisons to avoid overly conservative type II errors. The findings should be interpreted as generating hypotheses for future validation.

## 3 RESULTS

### 3.1 Participants’ Characteristics

A total of 32 post-stroke patients (24 males and 8 females; mean age 65.4 ± 10.2 years) and 27 HCs (16 males and 11 females; mean age 65.0 ± 4.7 years) were finally included in this study. The demographic characteristics and cognitive data for all participants are presented in Table **1**. There were no significant differences in age, sex, years of education, or TIV between the two groups (all *p* > 0.05). Compared to HCs, post-stroke patients exhibited poorer performance on all cognitive tests (all *p* < 0.001).

### 3.2 Differences in Glymphatic Markers between the Post-Stroke Patients and the HCs

Compared to the HCs group, the post-stroke patients group showed a significant decrease in the DTI-ALPS index (*p* < 0.001) and a significant increase in CPV (*p* < 0.001). However, no significant difference was observed in PVS volume between the two groups (Table **2**).

### 3.3 Association between Glymphatic Markers and Cognitive Function

**Fig. (4):**
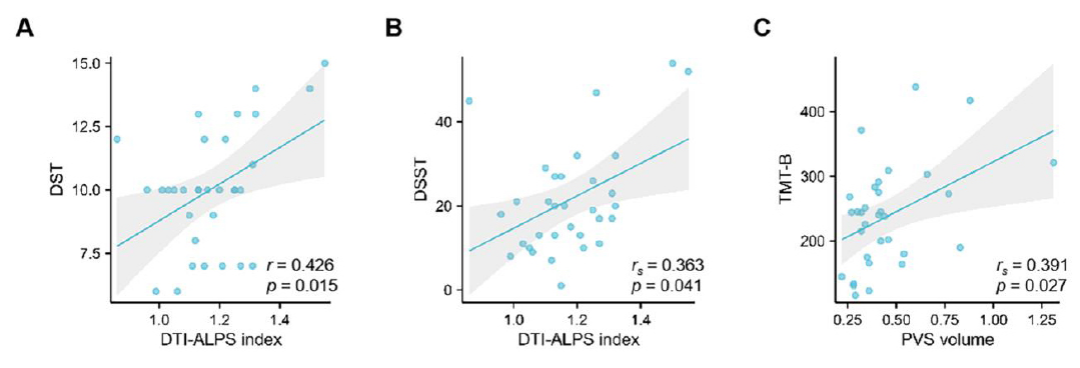
Correlations between glymphatic markers and cognition. The DTI-ALPS index was correlated to DST (**A**) and DSST (**B**). PVS volume was correlated to TMT-B (**C**). DTI-ALPS, diffusion tensor image analysis along the perivascular space; PVS, perivascular spaces. DST, digit span test; DSST, digit symbol substitution test; TMT-B, trail making test-B.

Correlation analysis demonstrated that the DTI-ALPS was positively correlated with the DST (*r* = 0.426, *p* = 0.015) and DSST (*r_s_* = 0.363, *p* = 0.041) scores. Additionally, the PVS volume was positively correlated with the TMT-B scores (*r_s_* = 0.391, *p* = 0.027) (Fig. **4**). After adjusting for age and years of education, linear regression analysis showed that the PVS volume was an independent predictor of TMT-B performance (*β* = 0.428, *p* = 0.010) (Table **3**). The decreased DTI-ALPS index was associated with poorer performance in working memory and processing speed, while the enlarged PVS volume was associated with poorer executive function performance.

## 4 DISCUSSION

In this study, we measured three glymphatic markers (DTI-ALPS index, CPV, and PVS volume) to assess the glymphatic function and explore its correlation with cognitive function. We found that (1) glymphatic function was impaired in post-stroke patients, as indicated by the decreased DTI-ALPS index and increased CPV compared to HCs; (2) glymphatic dysfunction in patients was correlated with certain cognitive domains, including working memory, processing speed, and executive function.

Our study using *in vivo* imaging methods found a decreased DTI-ALPS index and increased CPV in post-stroke patients, indicative of functional alterations in the glymphatic system. Our results were consistent with the findings by Toh *et al*. [16], who observed a decreased DTI-ALPS index within 60 days post-stroke. However, their study also demonstrated an increase in the DTI-ALPS index over time, suggesting recovery of the glymphatic system. In contrast, our study found that the DTI-ALPS index remained significantly decreased at 6 months post-stroke, indicating persistent damage to the glymphatic system. Animal studies have demonstrated ischemic stroke to impair glymphatic function through multiple interconnected mechanisms. First, ischemic injury led to the depolarization of AQP-4 channels, disrupting polarized fluid transport critical for metabolic waste removal (*e.g*., amyloid-β, tau) and inflammatory mediator clearance [24, 25]. Second, ischemia-induced necrotic cell death released damage-associated molecular patterns, which activated microglia and astrocytes and triggered a proinflammatory cytokine cascade [26]. Furthermore, neutrophil extracellular traps and inflammation-mediated enlargement of the perivascular spaces impaired glymphatic transport [27]. This glymphatic dysfunction promoted the accumulation of neuroinflammatory mediators, creating a pathological feedback loop and establishing a vicious cycle of “glymphatic dysfunction-neuroinflammation” [28]. Moreover, cerebral hypoperfusion attenuated arterial pulsatility, the primary driving force for CSF-ISF exchange *via* glymphatic pathways [29]. Collectively, these mechanisms substantially compromised the efficiency of CSF-ISF exchange, constituting the core pathological basis for post-stroke cognitive impairment. The choroid plexus plays a pivotal role in regulating the production and composition of CSF. Emerging evidence suggests that choroid plexus dysfunction disrupts CSF homeostasis, consequently impairing the efficiency of the glymphatic system clearance [30]. Several studies have revealed increased CPV in AD, PD, and other neurological disorders, with demonstrated correlations between increased CPV and cognitive impairment [8-10, 31]. Interestingly, while CPV expansion has also been observed in post-stroke patients, we identified no significant correlation between CPV and cognitive function in our study.

Previous studies have demonstrated that glymphatic dysfunction in AD, cerebral small vessel disease, moyamoya disease, traumatic brain injury, and other conditions, is associated with cognitive decline [7, 32-35]. In our study, comprehensive assessments of cognitive domains were evaluated in post-stroke patients to investigate their correlations with glymphatic markers. The decreased DTI-ALPS index in post-stroke patients correlates with a decline in working memory (as assessed by DST) and processing speed (as assessed by DSST). Dysfunction of the glymphatic system impaired the clearance of neurotoxic metabolites, particularly amyloid-β and hyperphosphorylated tau proteins [36]. The accumulation of these toxic proteins can disrupt synaptic plasticity and trigger neuronal apoptosis [37, 38], which may subsequently lead to reduced working memory capacity and slowed information processing speed. Notably, our study did not detect significant differences in PVS volume between post-stroke patients and HCs. This outcome may be attributable to the following factors: firstly, the relatively small sample size of this study might have been insufficient to identify subtle intergroup differences in PVS volume. Secondly, the PVS volume analysis adopted in this study did not further distinguish the PVS changes in regions, such as the BG or DWM. Studies have shown that enlarged PVS in the BG is more strongly associated with hypertension and blood pressure variability, while enlarged PVS in the DWM is more strongly associated with AD and cerebral amyloid angiopathy [39]. Nevertheless, we observed an independent association between PVS volume and executive function (as assessed by TMT-B). This finding was consistent with the study by Andriuta *et al*. [13], which demonstrated PVS to be significantly associated with executive function mediated by neuroinflammatory markers (glial fibrillary acidic protein; GFAP) and white matter hyperintensities (WMH), thus highlighting the potential role of PVS in cognitive impairment. The DTI-ALPS index and PVS may serve as predictors of domain-specific cognitive decline in post-stroke patients, offering clinical potential for guiding rehabilitation strategies and the management of PSCI.

Although this study has provided novel insights into the relationship between glymphatic dysfunction and cognitive decline in post-stroke patients, several limitations should be acknowledged. First, ischemic stroke is a highly complex and heterogeneous disease. However, our study did not account for factors, such as infarct location and volume, which may differentially modulate glymphatic dysfunction and cognition. Second, the relatively limited sample size constrained our ability to stratify patients into clinically meaningful subgroups based on post-stroke cognitive status, such as normal cognition, mild cognitive impairment, or dementia. In the future, larger cohorts will be necessary to ensure statistical robustness. Third, the cross-sectional design precluded causal inferences regarding the relationship between glymphatic dysfunction and cognitive impairment. Longitudinal studies are, therefore, warranted to track changes in glymphatic markers and their corresponding cognitive trajectories from the acute to chronic phases of stroke, thereby establishing temporal relationships and dynamic patterns. Fourth, in the analysis of PVS volume, we did not perform regional quantification of BG and DWM due to the limitations of the automated segmentation algorithm employed, although these regions may exhibit distinct association patterns with specific cognitive domains. Fifth, although we excluded individuals with a history of significant neurological or cerebrovascular diseases, the lack of systematic brain MRI screening or comprehensive vascular risk assessment in the control group may have resulted in undetected subclinical cerebrovascular pathologies (*e.g*., asymptomatic lacunar infarcts or white matter hyperintensities) among some healthy controls. This could potentially attenuate intergroup differences, leading to a bias toward null results, underestimating the true extent of glymphatic system impairment in stroke patients. Future studies should implement more rigorous controls for healthy comparison groups to minimize confounding factors. Finally, the three glymphatic markers (DTI-ALPS index, CPV, and PVS volume) employed in this study require further validation for their utility in assessing glymphatic function.

## CONCLUSION

In conclusion, this study has demonstrated glymphatic dysfunction, as evidenced by a reduced DTI-ALPS index and increased CPV, to persist six months after the onset of ischemic stroke and be significantly associated with deficits in specific cognitive domains, including working memory, information processing speed, and executive function. These findings have provided strong support for the hypothesis that impaired glymphatic function may play a contributory role in the development of PSCI. To further clarify causal mechanisms and assess the potential of glymphatic imaging as a predictive biomarker for cognitive outcomes or as a guide for therapeutic interventions, future studies with larger sample sizes and longitudinal designs are warranted.

## Figures and Tables

**Fig. (2):**
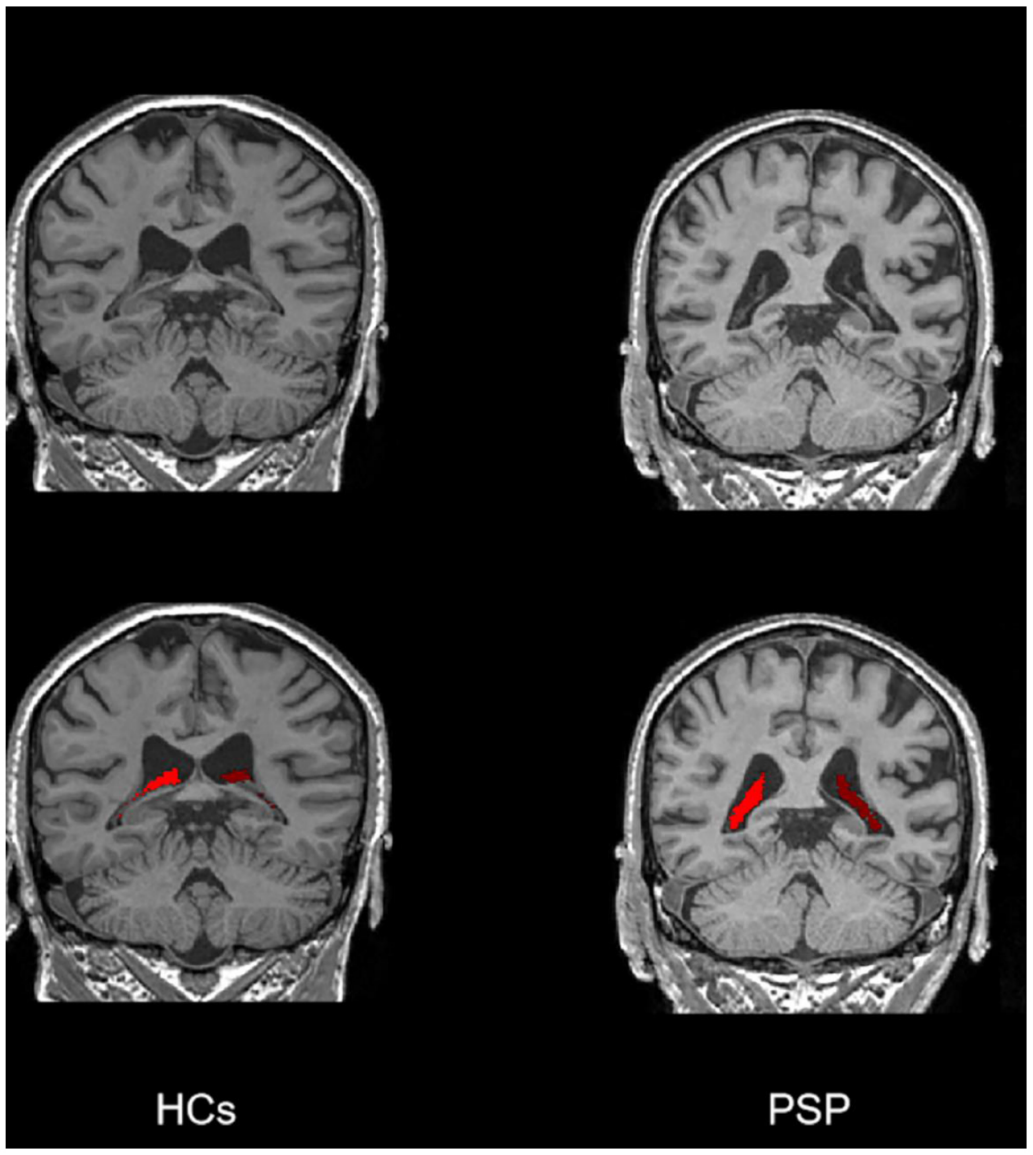
Comparison of the CPV segmentation on 3D-T1 images from the HCs group and the PSP group. The participants of both groups were 64-year-old females. CPV, choroid plexus volume; HCs, healthy controls; PSP, post-stroke patients.

**Fig. (3):**
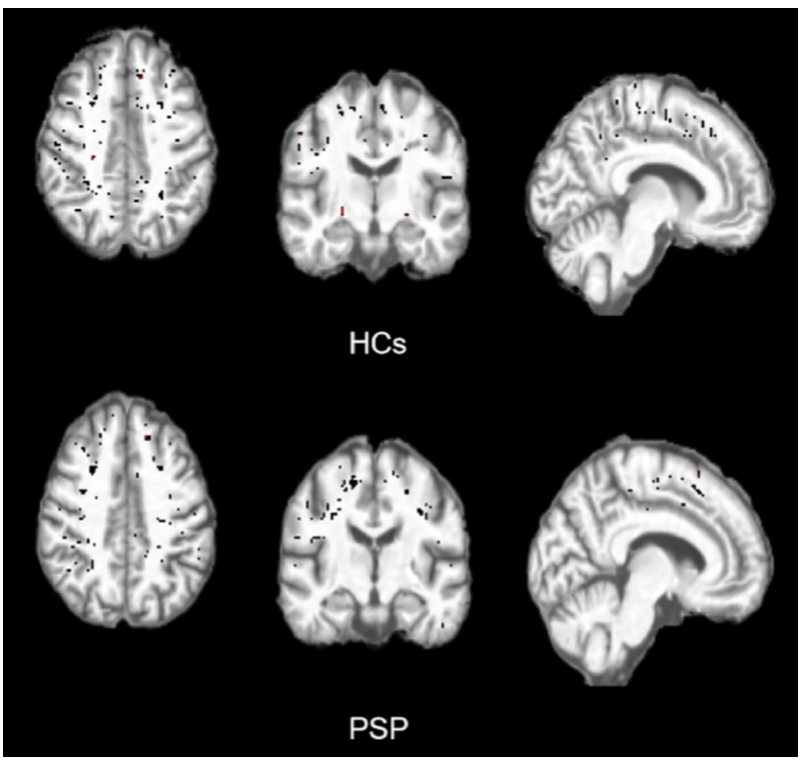
Comparison of the PVS volume segmentation on 3D-T1 images from the HCs group and the PSP group. The participants of both groups were 64-year-old females. PVS, perivascular spaces; HCs, healthy controls; PSP, post-stroke patients.

**Table 1:** Demographic characteristics and cognition between the PSP and HCs groups.

-	PSP (n = 32)	HCs (n = 27)	*p*-value
**Demographics**			
Age (years)	65.4 ± 10.2	65.0 ± 4.7	0.826
Male, n (%)	24 (75.0%)	16 (59.3%)	0.197
Education (years)	9.0 (7.0–9.3)	9.0 (7.0–10.5)	0.913
TIV (ml)	1462.51 (1401.73–1523.15)	1415.53 (1316.79–1488.26)	0.057
**Vascular risk factors**			
Hypertension, n (%)	24 (75%)	–	–
Diabetes, n (%)	11 (34.4%)	–	–
Hyperlipidemia, n (%)	6 (18.8%)	–	–
Hyperhomocysteinemia, n (%)	12 (37.5%)	–	–
Smoking, n (%)	14 (43.8%)	–	–
Drinking, n (%)	7 (21.9%)	–	–
Atrial fibrillation, n (%)	11 (34.4%)	–	–
**Cognition**			
MoCA	19.6 ± 4.7	26.0 ± 1.1	< 0.001
MMSE	23.8 ± 3.7	–	–
DST	10.1 ± 2.3	12.2 ± 1.7	< 0.001
DSST	19.5 (12.5–27.0)	35.2 ± 10.3	< 0.001
TMT-A	106.8 ± 41.2	64.4 ± 15.2	< 0.001
TMT-B	238.2 ± 79.8	171.4 ± 64.6	< 0.001
AVLT	13.3 ± 4.0	22.3 ± 6.2	< 0.001
AVLT-D	2.0 (0.0–4.0)	5.0 ± 2.6	< 0.001

**Note: **Results are expressed as mean ± standard deviation (SD) and median [interquartile range (IQR)]. **Abbreviations:** PSP, post-stroke patients; HCs, healthy controls; TIV, total intracranial volume; MoCA, Montreal cognitive assessment; MMSE, mini-mental state examination; DST, digit span test; DSST, digit symbol substitution test; TMT-A, trail making test-A; TMT-B, trail making test-B; AVLT, auditory verbal learning test; AVLT-D, auditory verbal learning test-delayed recall.

**Table 2:** Glymphatic markers of the PSP and HCs groups.

-	PSP (n = 32)	HCs (n = 27)	*p*-value
DTI-ALPS index, mean ± SD	1.18 ± 0.15	1.59 ± 0.21	< 0.001
CPV, median (IQR)	2.53 (1.95−2.72)	1.59 (1.28−1.91)	< 0.001
PVS volume, median (IQR)	0.40 (0.32−0.47)	0.38 (0.32−0.45)	0.626

**Note: **PSP, post-stroke patients; HCs, healthy controls; DTI-ALPS, diffusion tensor image analysis along the perivascular space; CPV, choroid plexus volume; PVS, perivascular spaces. The CPV (ml)/TIV (ml)*10^3^ is used to represent CPV; the PVS volume (ml)/TIV (ml)*10^3^ is used to represent PVS volume.

**Table 3:** Multiple linear regression analysis among DTI-ALPS index, PVS volume, and cognitive function.

Cognitive Scale	Variable	*β*	95% CI	VIF	*p*-value
DST	DTI-ALPS index	0.254	-0.154−0.661	1.478	0.213
DSST	DTI-ALPS index	0.125	-0.246−0.495	1.478	0.496
TMT-B	PVS volume	0.428	0.112−0.744	1.001	0.010

**Note: **DTI-ALPS, diffusion tensor image analysis along the perivascular space; PVS, perivascular spaces. DST, digit span test; DSST, digit symbol substitution test; TMT-B, trail making test-B.

## Data Availability

The data and supportive information are available within the article.

## LIST OF ABBREVIATIONS

AD: Alzheimer's disease
AQP-4: Aquaporin-4
AVLT: Auditory verbal learning test
BG: Basal ganglia
CPV: Choroid plexus volume
CSF: Cerebrospinal fluid
DSST: Digit symbol substitution test
DST: Digit span test
DTI-ALPS: Diffusion tensor image analysis along the perivascular space
DWM: Deep white matter
HCs: Healthy controls
ISF: Interstitial fluid
IQR: Interquartile range
MMSE: Mini-mental state examination
MoCA: Montreal cognitive assessment
PD: Parkinson’s disease
PSCI: Post-stroke cognitive impairment
PSP: Post-stroke patients
PVS: Perivascular spaces
SCR: Superior corona radiata
SD: Standard deviation
SLF: Superior longitudinal fasciculus
TIV: Total intracranial volume
TMT: Trail making test

## STANDARDS OF REPORTING

STROBE guidelines were followed.
